# A Comparative Analysis of Retrieval Algorithms of Land Surface Temperature from Landsat-8 Data: A Case Study of Shanghai, China

**DOI:** 10.3390/ijerph18115659

**Published:** 2021-05-25

**Authors:** Yue Jiang, Wenpeng Lin

**Affiliations:** School of Environment Science and Geography, Shanghai Normal University, Shanghai 200234, China; 1000496184@smail.shnu.edu.cn

**Keywords:** Landsat-8, land surface temperature retrieval, split-window algorithm, Mono-Window Algorithm, radiative transfer equation, Single-Channel Algorithm

## Abstract

In the trend of global warming and urbanization, frequent extreme weather has a severe impact on the lives of citizens. Land Surface Temperature (LST) is an essential climate variable and a vital parameter for land surface processes at local and global scales. Retrieving LST from global, regional, and city-scale thermal infrared remote sensing data has unparalleled advantages and is one of the most common methods used to study urban heat island effects. Different algorithms have been developed for retrieving LST using satellite imagery, such as the Radiative Transfer Equation (RTE), Mono-Window Algorithm (MWA), Split-Window Algorithm (SWA), and Single-Channel Algorithm (SCA). A case study was performed in Shanghai to evaluate these existing algorithms in the retrieval of LST from Landsat-8 images. To evaluate the estimated LST accurately, measured data from meteorological stations and the MOD11A2 product were used for validation. The results showed that the four algorithms could achieve good results in retrieving LST, and the LST retrieval results were generally consistent within a spatial scale. SWA is more suitable for retrieving LST in Shanghai during the summer, a season when the temperature and the humidity are both very high in Shanghai. Highest retrieval accuracy could be seen in cultivated land, vegetation, wetland, and water body. SWA was more sensitive to the error caused by land surface emissivity (LSE). In low temperature and a dry winter, RTE, SWA, and SCA are relatively more reliable. Both RTE and SCA were sensitive to the error caused by atmospheric water vapor content. These results can provide a reasonable reference for the selection of LST retrieval algorithms for different periods in Shanghai.

## 1. Introduction

Continued urbanization leads to a deterioration of the urban thermal environment and serial ecological consequences. Affected by the thermal environment, the morbidity and mortality from heat stress and heat-related diseases have increased, threatening the health and lives of citizens [1,2,3]. Land Surface Temperature (LST) retrieval is the most common method used for studying the effects of urban heat islands. LST is one of the most important parameters for characterizing land surface energy balance and water cycle processes at both regional and global scales, which is also essential in the hydrological cycle and climate prediction. It is widely used in various fields, including natural disaster monitoring and prevention, heat island effect assessment, crop yield estimation, vegetation monitoring, climate changes, etc. [4,5,6,7] The conventional method of obtaining LST is to use measured data from meteorological stations, which has the advantages of high reliability and long-term data availability. However, as these provide point temperature with limited spatial coverage, the data are often sparsely or irregularly distributed and cannot provide accurate LSTs on a large spatial scale. Nevertheless, the rapid development of remote sensing techniques has enabled satellite images to acquire near-real-time data on detailed spatial surfaces. Therefore, to obtain high-resolution, consistent, and repetitive LST at the regional and global scales, remote sensing is the only practical and feasible way [8,9,10,11,12]. Many satellites carry thermal infrared sensors, such as MODIS/Terra and Aqua, advanced very high-resolution radiometer (AVHRR)/NOAA, and Landsat-8/TIRS [13]. Landsat-8 was launched on 11 February 2013, and put into orbit with two instruments onboard: the Operational Land Imager (OLI) and the Thermal Infrared Sensor (TIRS) [14]; the Landsat-8 has two TIRS bands in the atmospheric window between 10 and 12 μm, whereas Landsat TM/ETM+ sensors only have one thermal band. The two TIRS bands (band 10 and band 11) with similar bandwidths and center wavelengths to the MODIS sensor band 31 and band 32, with a spatial resolution of 100 m, make atmospheric corrections easier and provide the possibility of SWA. Therefore, the data from the Landsat-8 are more advantageous in LST retrieval studies than those from previous satellite remote sensing products of the same series and are more suitable for quantitative studies and precise analysis of retrieved LST.

Scholars worldwide have proposed various LST retrieval algorithms from satellite images, which include the Mono-Window Algorithm (MWA), Radiative Transfer Equation (RTE), Split-Window Algorithm (SWA), Single-Channel Algorithm (SCA), Multi-Angle Algorithm, Multi-Channel Algorithm, and Hyperspectral Algorithm [15,16,17,18,19,20]. These LST retrieval algorithms require several corresponding parameters, including the land surface emissivity (LSE), effective mean atmospheric temperature, and atmospheric transmittance. The influence of the diversity of retrieval algorithms and their associated parameters makes it difficult for scholars in other non-specialized fields to quickly and accurately select the appropriate algorithm for LST retrieval. In order to provide a valuable reference for the selection of LST retrieval algorithms for different periods in Shanghai, this study analyzed and compared the accuracy of retrieval results of the RTE, MWA, SWA, and SCA, testing the suitability of these algorithms for LST retrieval by using the Landsat-8 remote sensing data in winter and summer.

## 2. Study Area and Data

### 2.1. Study Area

**Figure 1:**
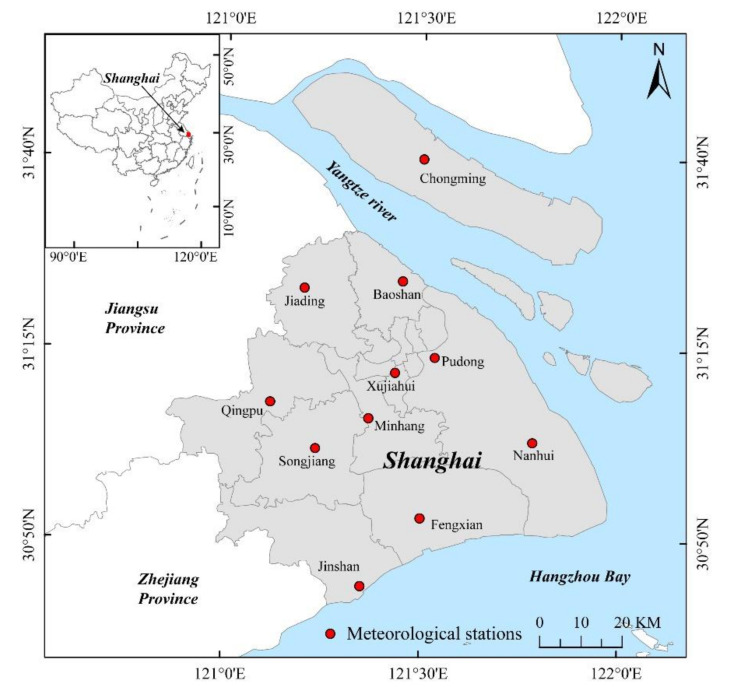
The location of the study area and meteorological stations.

This study used the administrative territory of Shanghai (see Figure 1) as the study area. Shanghai is located on the west coast of the Pacific Ocean and the eastern edge of the Asian continent. The city is also situated in the confluence area of the Yangtze River and the Huangpu River as they flow into the East China Sea. The Yangtze River bounds shanghai to the north, the East China Sea to the east, Hangzhou Bay to the south, and Jiangsu and Zhejiang provinces to the west. The area’s climate is a subtropical monsoon climate with four distinct seasons, long hours of sunshine, and abundant precipitation. The study area covers 16 districts with a land area of 6340 km^2^ and is located between latitudes 30°40′ N and 31°53′ N, and longitudes 120°52′ E and 122°12′ E. Shanghai has been identified as one of China’s rapidly developing cities; it is an important economic, scientific, and comprehensive transportation center. In the process of rapid urbanization, the city is facing some environmental issues such as the urban heat island effect.

### 2.2. Data Resources

In this study, Landsat data were used as the primary source of data. The Landsat-8 satellite images for the winter (4 December 2019, 21 January 2020, and 22 February 2020) and summer (29 July 2019, 12 May 2020, and 16 August 2020) were obtained from the United States Geological Survey (USGS) earth explorer website (https://www.usgs.gov/, (accessed on 22 May 2021)) [21]. The LST retrieval results were analyzed and compared with the temperatures of local meteorological stations obtained from the China Meteorological Data Network (https://data.cma.cn/) [22], thus validating the study results. Besides performing a cross-validation of the results with MODIS daily LSTs, the study downloaded the MODIS LST product (MOD11A2) from NASA’s official website (https://ladsweb.nascom.nasa.gov/, (accessed on 22 May 2021)) [23]. The SWA needed to estimate atmospheric transmittance by water vapor content obtained from the TERRA/MODIS water vapor product MOD05, which was obtained from NASA’s official website. The study’s ancillary data included Land Cover (LC) from the GlobeLand30 (http://www.globallandcover.com/, (accessed on 22 May 2021)) [24].

### 2.3. Data Preprocessing

The Landsat-8 images used were already orthorectified, so no geo-correction was needed during preprocessing. Radiometric calibration, atmospheric correction, and image cropping were all part of the data preprocessing. To obtain accurate information on the radiation received by the sensors, the digital number (DN) values of Landsat-8 data were converted to emissivity by radiometric calibration. Besides eliminating atmospheric and illumination factors from the sensors’ radiation value and identifying the actual land surface emissivity (LSE), the atmospheric correction of data was needed to reflect the feature information accurately. The radiometric calibration and atmospheric correction were performed using the ENVI software (version 5.3) (Exelis Visual Information Solutions, Boulder, CO, USA).

## 3. Methodology

### 3.1. Parameters for Retrieving Land Surface Temperature

#### 3.1.1. Brightness Temperature

The brightness temperature corresponds to the temperature of a black body having the same intensity of radiation as the observed object. It is a measure of the object’s temperature, but not the actual temperature of the object. To estimate the brightness temperature, the DN value of each pixel is first converted into the spectral energy using the given calibration values, and then the spectral radiance is converted to brightness temperature by the Planck radiation function.

(1) $$T_{i}=\frac{K_{2}}{\ln\left(K_{1}/L_{\lambda }+1\right)}$$

where *L_λ_* is spectral radiation (W·m^−2^·sr^−1^·μm^−1^); *T_i_* is the recorded brightness temperature on the sensor surface (K) corresponding to the band *i* (*i* = 10, 11); *K*_1_ and *K*_2_ are the constant coefficients that vary for different Landsat satellites. The *K_1_* values in bands 10 and 11 of the Landsat-8 satellite are estimated at 774.89 and 480.89 W·m^−2^·sr^−1^·μm^−1^, respectively, while the *K_2_* values in these bands are 1321.08 and 1201.14 W·m^−2^·sr^−1^·μm^−1^, respectively.

#### 3.1.2. Land Surface Emissivity

The land surface emissivity (LSE) is an essential parameter for retrieving LST. The LSE indicates the actual ability of an object to emit energy relative to a black object with the same temperature. Different emissive materials have different emissivity. It depends on their different surface materials, surface roughness, vegetation cover, and even the wavelength interval of the remote sensing sensor’s electromagnetic waves. For the Landsat-8 image data, this study estimated LSE with the normalized difference vegetation index (*NDVI*)-based emissivity method (NBEM). The principle of the *NDVI* thresholding method is to classify the pixels of the image into three categories—pure vegetation pixels, bare soil pixels, and mixed pixels—based on *NDVI* values at a certain threshold. The following equation can calculate the LSE of the mixed pixel, which was proposed by Qin et al.:

(2) $$F_{v}=\frac{NDVI-NDVI_{soil}}{NDVI_{Veg}-NDVI_{soil}}$$

(3) $$\varepsilon_{i}=F_{v}\varepsilon_{iv}+\left(1-F_{v}\right)\varepsilon_{is}$$

where *F_v_* is the fractional vegetation cover; *NDVI_veg_* is the *NDVI* of pure vegetation pixels; *NDVI_soil_* is the *NDVI* of pure bare soil pixels. When 0 < *NDVI* < *NDVI_soil_*, the pixel can be treated as bare soil with LSE values of 0.971 for band 10, and 0.977 for band 11. When *NDVI* > *NDVI_veg_*, the pixel can be considered pure vegetation with LSE values of 0.987 and 0.989 for band 10 and band 11, respectively. When *NDVI_soil_* ≤ *NDVI* ≤ *NDVI_veg_*, the pixel can be regarded as mixed pixels, and the LSE value can be calculated according to Formula (3). *ε_i_* is the LSE in band *i* (*i* = 10, 11); *ε_iv_* and *ε_is_* are the vegetation and bare soil emissivity of band i, respectively.

#### 3.1.3. Atmospheric Transmittance

In the four LST retrieval algorithms, atmospheric transmittance is an important parameter, and the variation of atmospheric water vapor content directly affects the calculation of atmospheric transmittance. Since NASA’s atmospheric correction parameter calculator only publishes the atmospheric profile data of band 10 of the Landsat-8, and SWA requires the atmospheric transmittance data of bands 10 and 11, this study used the MOD05L2 product that has the same date and the closest transit time to that of the Landsat-8 images in order to estimate the atmospheric water vapor content. This study subsequently calculated the atmospheric transmittance based on the linear relationship between atmospheric water vapor content and atmospheric transmittance, which could be expressed in band 10 and band 11 as: *τ_10_ = −*0.1134*∙W* + 1.0335 and *τ_11_ = −*0.1546*∙W* + 1.0078, respectively.

### 3.2. Four Methods of Retrieving LST from Landsat-8

#### 3.2.1. Radiative Transfer Equation

The Radiative Transfer Equation (RTE), also known as the atmospheric correction method, is based on the principle of subtracting the atmospheric influence on surface radiation from the total amount of thermal radiation observed by the satellite sensors in order to obtain the surface thermal radiation intensity, subsequently converting it to the corresponding LST. The Formula (4) can be expressed as:

(4) $$T_{S}=\frac{K_{2}}{\ln\left[1+K_{1}/B\left(T_{s}\right)\right]}$$

In the formula, $L_{\lambda }=\left[\varepsilon B\left(T_{s}\right)+\left(1-\varepsilon \right)L_{-}\right]\tau +L^{+}$,$B\left(T_{s}\right)=\left[L_{\lambda }-L^{+}-\tau \left(1-\varepsilon \right)L_{-}\right]/\tau \varepsilon$, where *L^+^* and *L_−_* are the upwelling and downwelling radiance in the atmosphere; *τ* is the average atmospheric transmittance in the thermal infrared band. Estimate the three parameters mentioned above by entering some specified parameters—including latitude, longitude, time, and date—into the online atmospheric correction calculator on NASA’s website (http://atmcorr.gsfc.nasa.gov/, (accessed on 22 May 2021)) [25] and by inputting some mandatory data including location (longitude and latitude), date, and time. ε is the Land Surface Emissivity (LSE). *B(T_s_)* is the radiation brightness received by the sensor for a blackbody with temperature *T_s_* (W·m^−2^·sr^−1^·μm^−1^). *Ts* is the land surface temperature (K). *K_1_* and *K_2_* are the constant coefficients that can be obtained from the Landsat-8 MTL file.

#### 3.2.2. Mono-Window Algorithm

Qin et al. proposed the Mono-Window Algorithm (MWA) based on the radiative transfer equation for the Landsat TM band 6 data, which can avoid real-time dependence data. However, it is only applicable to satellite images with only one thermal infrared channel. Fei Wang et al. [26] improved the algorithm and made it suitable for band 10 of Landsat-8. It can be expressed as the following Formula (5):

(5) $$T_{s}=\frac{a\left(1-C-D\right)+\left(b\left(1-C-D\right)+C+D\right)T_{b}-DT_{a}}{C}$$

In the formula, C=ετ,$D=\left(1-\tau \right)\left[1+\left(1-\tau \right)\tau \right]$; *ε* is the LSE and *τ* is the atmospheric transmittance; *T_s_* is LST (K); *a* and *b* are constants—in summer, *a* = −70.1775, b = 0.4581, and in winter, *a* = −55.4276, *b* = 0.4086. *T_b_* is the at-sensor brightness temperature; *T_a_* is the effective mean atmospheric temperature; *T_0_* is the near-surface air temperature (K), obtained from the historical weather website (http://weather.bsyan.com/) [27]. There is a linear relationship between *T_0_* and *T_a_* at different conditions; in summer, the effective mean atmospheric temperature can be calculated by $T_{a}=16.0110+0.92621T_{0}$, and in winter, the equation can be expressed as $T_{a}=19.2704+0.91118T_{0}$.

#### 3.2.3. Split-Window Algorithm

McMillin et al. initially proposed a split-window algorithm for observing ocean surface temperature based on AVHRR thermal infrared data [28]. Rozenstein et al. and Qin et al. improved the SWA based on the work of previous scholars [29]. The specific calculation formula is shown below:

(6) $$T_{s}=A_{0}+A_{1}T_{10}-A_{2}T_{11}$$

where *T_s_* is the LST (K). T_10_ and T_11_ are the brightness temperature of band 10 and band 11. *A_0_*, *A_1_*, and *A_2_* are algorithm coefficients ($A_{0}=E_{1}a_{10}-E_{2}a_{11}$,$A_{1}=1+A+E_{1}b_{10}$,$A_{2}=A+E_{2}b_{11}$); *A*, *E*_0_, *E*_1_, and *E*_2_ are intermediate parameters determined by emissivity and atmospheric transmittance ($E_{0}=D_{11}C_{10}-D_{10}C_{11}$,$E_{1}=[D_{11}\left(1-C_{10}-D_{10}\right)]/E_{0}$,$E_{2}=[D_{10}\left(1-C_{11}-D_{11}\right)]/E_{0}$,$\;A=D_{10}/E_{0}$), where $C_{i}=\varepsilon_{i}\tau_{i}$, $D_{i}=\left(1-\tau_{i}\right)\left[1+\left(1-\tau_{i}\right)\tau_{i}\right]$, *ε_i_* and *τ_i_* are the LSE and the atmospheric transmittance of TIRS band 10 and 11, respectively. *a*_10_, *b*_10_, and *a*_11_, *b*_11_ are the empirical coefficients, which need to consider the location of the study area and the season of image acquisition. Since the winter land surface temperature range in Shanghai is between 0 and 30 °C, *a*_10_ = −59.1391, *b*_10_ = 0.4213 and *a*_11_ = −63.3921, *b*_11_ = 0.4565, and the summer temperature range is between 10 and 50 °C, *a*_10_ = −64.6081, *b*_10_ = 0.4399 and *a*_11_ = −69.0215, *b*_11_ = 0.4756.

#### 3.2.4. Single-Channel Algorithm

The Single-Channel Algorithm (SCA) was developed by Jimenez-Munoz and Sobrino. It is widely used because it requires few real-time atmospheric parameters and only the LSE and the atmospheric water vapor contents; therefore, the final retrieval error due to effective mean atmospheric temperature can be reduced. This algorithm was later adapted for Landsat-8 data to retrieve LST, and the specific formula can be expressed as:

(7) $$T_{s}=\gamma \left[\frac{1}{\varepsilon }\left(\psi_{1}L_{sen}+\psi_{2}\right)+\psi_{3}\right]+\delta$$

where *T_s_* is the LST (K). *ε* is LSE; *ψ*_1_, *ψ*_2_, *ψ*_3_ are atmospheric function parameters (*ψ*_1_ *= 1/τ*; *ψ*_2_ *= −L_−_ − L^+^/τ*; *ψ_3_ = L_−_*), *L^+^* and *L_−_* are the upwelling and downwelling radiance in the atmosphere, and *τ* is the atmospheric transmittance. *γ* and *δ* are the intermediate parameters ($\gamma \approx T_{sen}^{2}/b_{\gamma }L_{sen}$;$\;\delta \approx T_{sen}-T_{sen}^{2}/b_{\gamma }$), *T_sen_* is the at-sensor brightness temperature of TIRS band 10; *L_sen_* is the at-senor registered radiance (W·m^−2^·sr^−1^·μm^−1^), which can be obtained after radiometric calibration; *b_γ_ = c*_2_*/λ*, *λ* is the effective wavelength of TIRS, and the value of *b_γ_* in band 10 is taken as 1324 K.

## 4. Results

### 4.1. Distribution Analysis of LST

**Figure 2:**
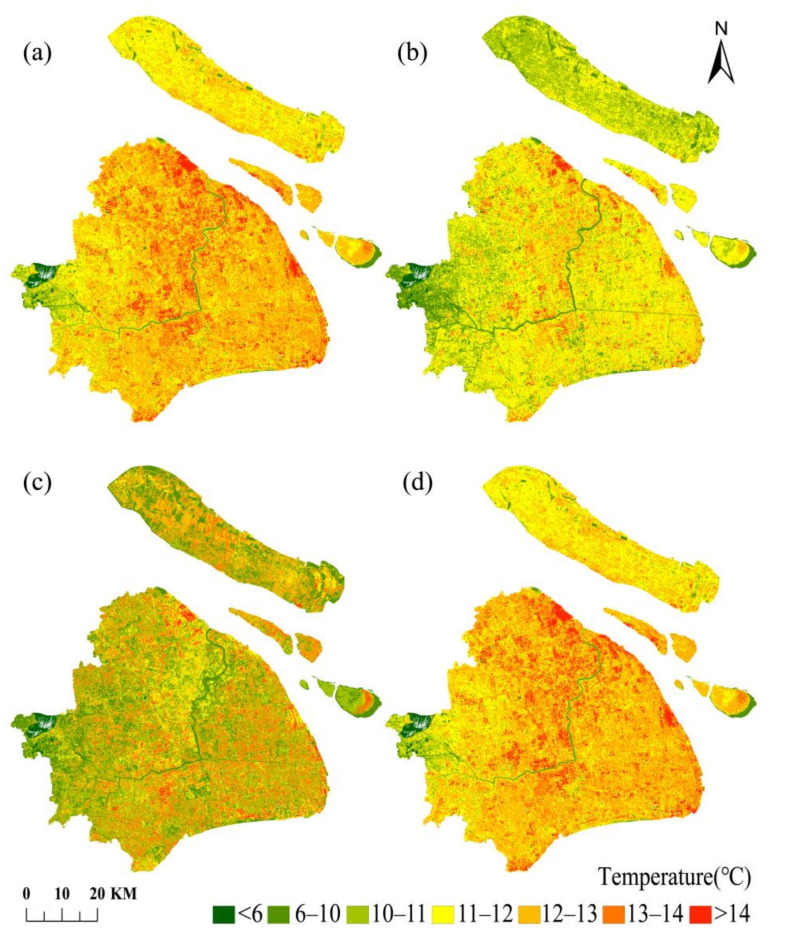
Comparison of the retrieval results of four algorithms on 21 January 2020. (**a**–**d**) are RTE, MWA, SWA, and SCA retrieval results, respectively).

**Figure 3:**
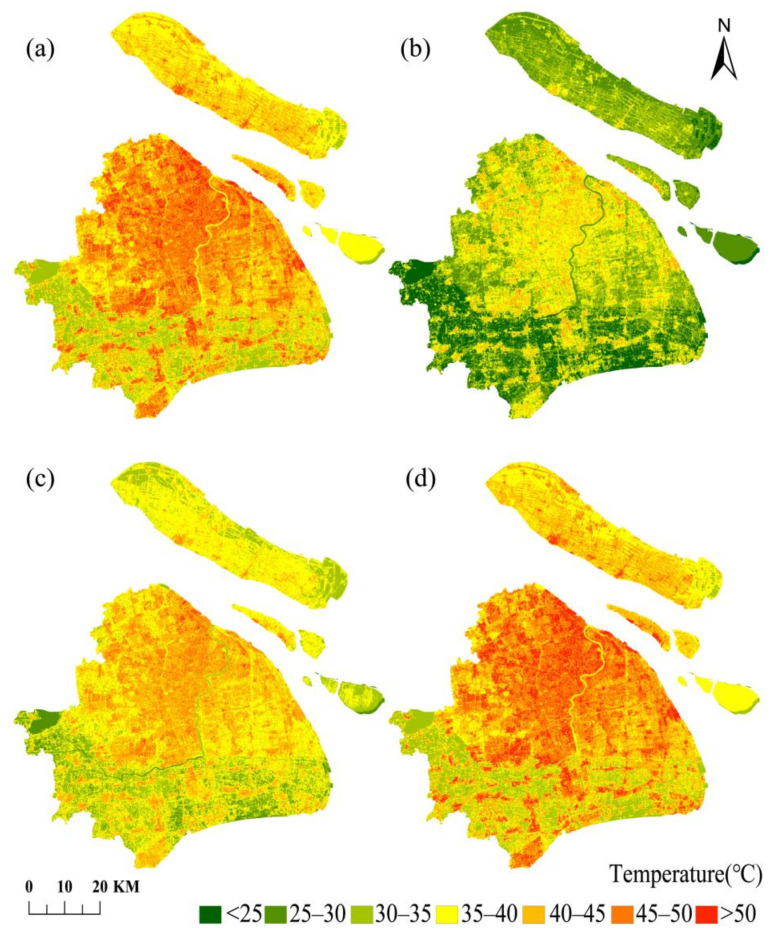
Comparison of the retrieval results of four algorithms on 16 August 2020. (**a**–**d**) are RTE, MWA, SWA, and SCA retrieval results, respectively.

The retrieved LST maps from the Landsat-8 TIRS data for winter (4 December 2019, 21 January 2020, and 22 February 2020) and summer (29 July 2019, 12 May 2020, and 16 August 2020) were taken using four LST retrieval algorithms, namely RTE, MWA, SWA, and SCA, shown in Figure 2 and Figure 3. The LST retrieval results for winter and summer are shown for 21 January 2020, and 16 August 2020.

From Figure 2 and Figure 3, we can observe that the general trend of land surface temperature obtained by the inversion of the four algorithms is generally the same. The LST in the central city is significantly higher than that in the suburbs of the city. The central city is of a high-temperature concentrated contiguous distribution, and the urban heat island effect is pronounced. This may be due to the high absorption rate of solar radiation by the impermeable subsurface in the city. In addition, the high concentration of population and the release of large amounts of heat from human production activities is coupled with the tall and dense buildings in the city, which seriously impede air circulation and are not conducive to heat exchange. These three reasons together lead to the city’s higher temperature. The areas with lower temperatures are mainly distributed with higher vegetation cover or located near water bodies. As is well known, increasing the vegetation cover in cities has a significant effect on regulating the urban microclimate, improving urban thermal development, and reducing the urban heat island effect. The four different algorithms obtained similar LST results, which indicate that all four algorithms can retrieve LST well. The results of all four algorithms can be used to study the urban heat island effect. These four surface temperature inversion algorithms achieved good overall results in studying the urban heat island effect.

**Figure 4:**
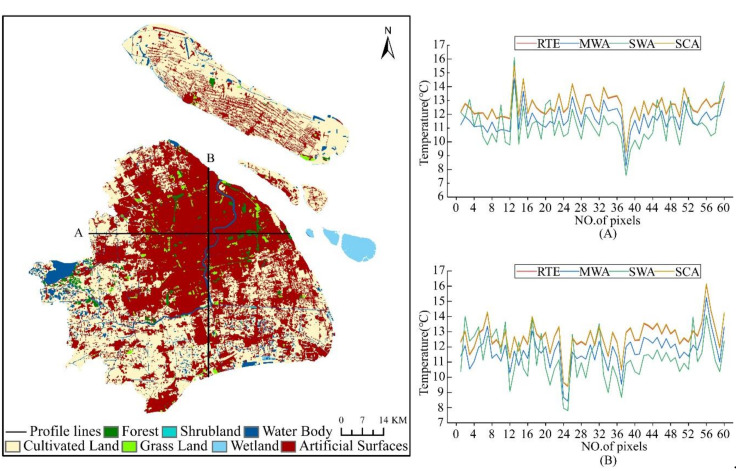
The land cover types in shanghai and the LST profile lines on 21 January 2020. (**A**) is the LST profile line in the east–west; (**B**) is the LST profile line in the south–north.

The profile analysis can visually reflect the characteristics and overall trends of the urban thermal environment. The selected profile must be typical, and the area it passes through can represent the general trend of the study area. To further analyze the spatial distribution pattern of the heat island effect in Shanghai, this study conducted a profile analysis based on the retrieval results of Landsat-8 images on 21 January 2020. The profile lines are centered on Shanghai People’s Square and extend in the east–west and south–north directions throughout Shanghai to obtain the LST profiles. The results are shown in Figure 4.

The east–west profile length is about 66.08 km, starting from Qingpu District in the west and ending in Pudong New District in the east, passing through Suzhou River, the urban core areas, and Huangpu River. From east to west, the main land cover types of the profile that were drawn from the figure were at the order of the suburban-urban regions, with impervious surfaces connected in the urban area, a single land cover type, high surface temperature, and a relatively gentle profile. Additionally, the lower temperatures in the western suburbs clearly show the existence of the heat island effect in Shanghai. The south–north profile is about 68.87 km in length, starting from the Baoshan industrial district in the northern suburbs, passing through the urban core areas and the Huangpu River, to Fengxian District in the south. The profile line passes through different land cover types, resulting in a large LST profile dithering amplitude, closely related to Shanghai’s urban development. It can be seen from the figure that the LSTs in the northwest regions are higher than other areas, and the comparison between these regions and the land cover map reveals that the regions are densely built-up areas in the urban center, mainly artificial surfaces, with a high urbanization level; human activities and natural factors caused the changes in some feature types. The southern suburbs are less urbanized and had alternating land cover types such as cultivated land, resulting in lower LSTs and larger amplitudes of the temperature profile line than in the north. Besides, several apparent low values in the profile corresponded to the Huangpu River area’s vicinity by positioning, further verifying that the urban water bodies have a cold island effect.

It is demonstrated that all four algorithms can better reflect the heat island effect in Shanghai by combining the spatial distribution of LST and the LST profiles. The temperature profile line dithering of SWA is more intense and has a higher amplitude compared to the other three algorithms, which can better reflect the differences in urban thermal environment changes due to different sub-bedding surface structures, and the difference between the artificial surfaces and other areas are larger in LST retrieval results. While RTE, MWA, and SCA’s temperature profile lines have roughly the same trend, RTE had better retrieval for the water body regions.

### 4.2. Comparison of LST Obtained by Four Algorithms

**Figure 5:**
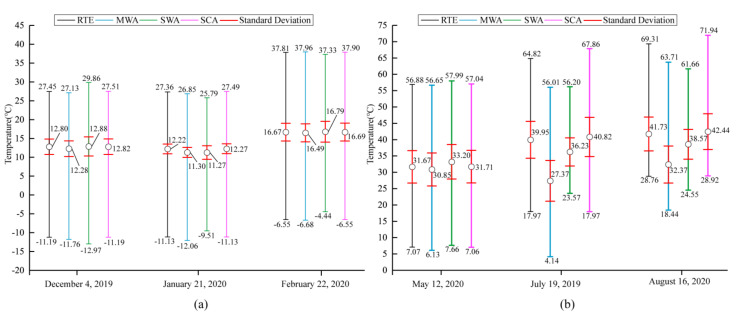
The statistical map of LST retrieval results of four algorithms in winter. (**a**) are the statistics results of LSTs in winter. (**b**) are the statistics results of LSTs in summer.

Based on the Landsat-8 images, the above Radiative Transfer Equation (RTE), Mono-Window Algorithm (MWA), Split-Window Algorithm (SWA), and Single-Window Algorithm (SCA) were used to retrieve LST. The differences between the four algorithms were explored by evaluating and comparing the retrieval results. Figure 5 shows the statistical plots of the four statistical indicators of minimum, maximum, standard deviation, and average values of the four algorithms’ retrieval results in winter and summer. Table 1 shows the differences (in absolute values) in the average LST values between the four algorithms, for any two algorithms. It can be seen that the overall trends of the surface temperature retrieval results of the four algorithms in different periods were relatively similar, but there were also some differences.

Figure 5 shows the statistical results of the four algorithms for LST retrieval in winter. The highest average LST was 16.66 °C in February, followed by 12.70 °C in December, and the lowest LST was 11.77 °C in January. The two algorithms with the largest difference in average values on 4 December were SWA and MWA, with a difference of 0.60 °C between them, followed by 0.54 °C between SCA and MWA, and the smallest difference of only 0.02 °C between SCA and RTE. The maximum difference between the average values on 21 January was 1.00 °C between SCA and SWA, and only 0.05 °C between SCA and MWA. The largest difference between the mean values on 22 February was 0.30 °C for SWA and MWA, and the smallest difference was only 0.02 °C for SCA and RTE. Overall, the LSTs obtained by RTE and SCA were the closest, with an average difference of only 0.03 °C. It can probably be attributed to the fact that both algorithms use the same simultaneous atmospheric parameters (atmospheric transmittance in the thermal infrared bands), and that the error between RTE and SCA was small, although the algorithms are different. The largest average temperature difference was 0.57 °C between MWA and SCA, followed by 0.54 °C between MWA and RTE, which was due to the error caused by introducing the average atmospheric temperature MWA to approximate the upwelling and downwelling radiation in the atmosphere. The average difference between SWA and the other three algorithms was between 0.31 and 0.39 °C, related to its need to use two thermal infrared bands.

The LST retrieval comparison of the four algorithms in the summer is shown in Figure 5. The maximum LST value obtained by SCA was higher than the three other algorithms. The minimum LST value obtained by MWA, by comparison, was lower than the three other algorithms. The average LST reached a height of 38.78 °C in August, followed by 36.09 °C in July, and 31.86 °C was the lowest average LST in May. On 12 May, the largest difference between the average values was 2.35 °C for SWA and MWA, followed by 1.51°C for RTE and SWA, and the smallest difference was just 0.04 °C for SCA and RTE. The largest difference between the average values of 29 July was between SCA and MWA, followed by RTE and MWA, and at only 0.87 °C the smallest difference was between SCA and RTE. The largest difference between the average values was between SCA and MWA on 16 August, followed by RTE and MWA, while the smallest difference was between SCA and RTE at just 0.71 °C.

With an average difference of only 0.54 °C, the error between the LST results obtained by RTE and SCA was the lowest. The maximum value of the average surface temperature difference between MWA and SCA was 8.1 3°C, followed by 7.59 °C between MWA and RTE, since the atmospheric water vapor content was more affected by MWA, and the water vapor content was higher and more variable in summer than in winter, leading to a higher error. Between SWA and the other three algorithms, the average difference was between 2.80 and 5.80 °C.

### 4.3. Validation of Retrieved LST

The high sensitivity of LST to spatial–temporal variations often makes it difficult to assess the accuracy and validation of satellite pixel-sized data. There are usually three methods to verify the LST retrieval results: Temperature-based method (T-based), Radiance-based method (R-based), and Cross-validation method. This study used the T-based validation method and the Cross-validation method to validate the retrieval result and evaluate the four algorithms’ accuracy.

#### 4.3.1. Evaluate T-Based Validation Results

**Figure 6:**
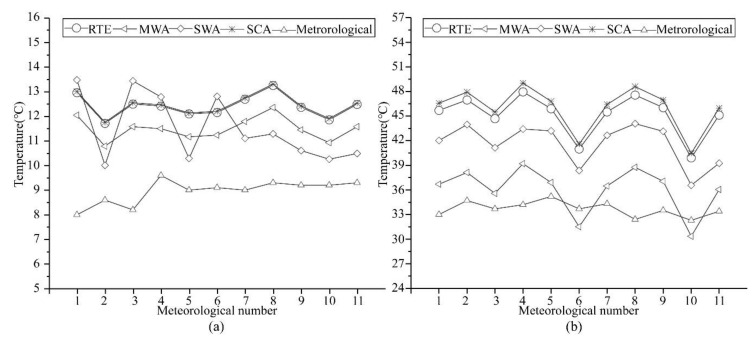
Comparison of local meteorological data with the retrieval results of four algorithms. (**a**) are the LST retrieval results compared with meteorological stations data on 21 January 2020; (**b**) are the LST retrieval results compared with meteorological stations data on 16 August 2020.

T-validation was performed, with data from the meteorological stations on 21 January 2020 and 16 August 2020 used as reference values for LST comparison. The comparison results are shown in Figure 6. On 21 January 2020, the average temperature differences between the RTE, MWA, SWA, and SCA retrieval results and the air temperature measured at the meteorological stations were 3.46 °C, 2.54 °C, 2.55 °C, and 3.51 °C, respectively. On 16 August 2020, the average temperature differences were 11.45 °C, 2.39 °C, 7.95 °C, and 12.32 °C, respectively. According to related research, the LST retrieval results are usually about 6 k higher than the air temperature in summer and about 3 k higher than the air temperature in winter [30], proving the reliability of this study’s retrieval results. It can be seen from Figure 6 that the retrieval results of these four algorithms have the same distribution curve as the air temperature.

The results mentioned above indicated that all the four algorithms could retrieve LSTs for winter in Shanghai. In summer, the RTE and SCA had larger errors than the other methods and were not suitable for the retrieval of LSTs. Meanwhile, MWA and SWA were better choices.

#### 4.3.2. Evaluate Cross-Validation Results

**Figure 7:**
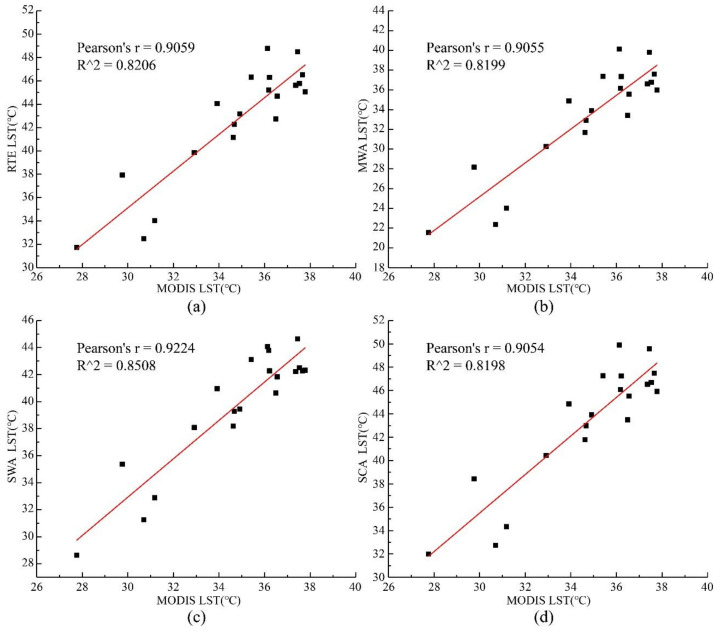
Comparison of land surface temperatures retrieved from Landsat-8 using four different algorithms with the MOD11A2 temperature product on 16 August 2020. (**a**) is the RTE; (**b**) is the MWA; (**c**) is the SWA; (**d**) is the SCA.

This paper utilized the MOD11A2 surface temperature product to perform the cross-validation of four algorithms. The cross-validation is advantageous because no field temperature measurement is needed, and the MODIS temperature products are easy to obtain. The MOD11A2 temperature product is based on the SWA’s retrieval results with a temporal resolution of 8 days and a spatial resolution of 1 km. Landsat-8 thermal infrared data’s spatial resolution is 100 m, and the MODIS products need to be resampled to 100 m in order to be consistent with retrieval results for comparison. On a homogeneous surface, MODIS and Landsat are on the same scale, and the surface temperature within the pure image element should be the same for both. The MOD11A2 temperature product was analyzed for consistency with the surface temperatures of the four algorithms by selecting different sample points at the same latitude and longitude location in the study area, as shown in Figure 7. The RTE, MWA, SWA, and SCA have an excellent linear curve relationship with MODIS products from an general perspective, with correlation coefficients of 0.8256, 0.8199, 0.8508, and 0.8198, and Pearson coefficients of 0.9059, 0.9055, 0.9224, and 0.9054, respectively.

**Figure 8:**
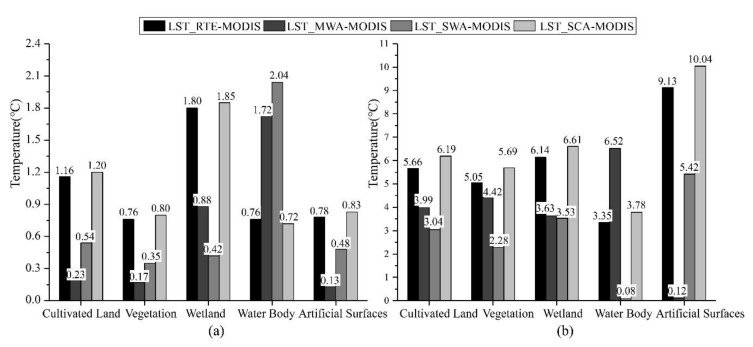
The average temperature difference between the retrieval results of four algorithms and the MOD11A2 surface temperature product under different land cover types. (**a**) are the statistics results of the average temperature difference on 21 January 2020; (**b**) are the statistics results of the average temperature difference on 16 August 2020.

Changes in urban location, the density of built-up areas, surface materials, and industrial activities can lead to dramatic LST changes. LST varies considerably between different types of land cover [31,32,33,34]. As mentioned previously, taking 21 January 2020 and 16 August 2020 as examples, the differences between the four algorithms were analyzed by calculating the average temperature difference on different land cover types with the MODIS products (taking absolute values). The results are shown in Figure 8.

As seen in Figure 8, in the winter of 21 January 2020, the largest error in the average temperature difference between the retrieval results of four Landsat-8 LST algorithms and the MOD11A2 product in the cultivated land was reached by SCA with 1.2 °C, followed by RTE with 1.16 °C, and SWA with 0.54 °C, while the smallest error was 0.23 °C for MWA. The mean temperature difference errors were ranked from largest to smallest as SCA, RTE, SWA, and MWA, consistent within the cultivated land in the vegetation and artificial surfaces. In the wetland, the RTE and SCA errors reached 1.8 °C, followed by 0.88 °C in MWA, while the SWA error was only 0.42 °C. However, in the water body, the largest mean temperature difference error was 2.04 °C for SWA, followed by 1.72 °C for MWA, 0.76 °C for RTE, and 0.72 °C for SCA. In the summer of 16 August 2020, SWA had the smallest average temperature difference from the MOD11A2 product, with an error range of 0.08 to 3.53 °C in all areas of cultivated land, vegetation, wetland, and water body, while the smallest average temperature difference was 0.12 °C in MWA on the artificial surfaces. The overall summer cross-validation results show that RTE and SCA have larger average temperature differences across the five land cover types than the other two algorithms. In contrast, SWA and MWA have smaller mean temperature differences from the MODIS temperature products.

As mentioned above, we can conclude the following: Firstly, the retrieval accuracy of the four LST algorithms is overall significantly higher in winter than in summer for different land cover types. Secondly, in the cultivated land, vegetation, and artificial surfaces in winter, MWA has the highest retrieval accuracy, followed by SWA, RTE, and SCA. In the wetland, SWA has the highest accuracy, followed by MWA, and the other two algorithms have lower accuracy. In contrast, in the water body, RTE and SCA have higher retrieval accuracy than the two other algorithms. Thirdly, in summer, the SWA has the highest retrieval accuracy for the area of cultivated land, wetland, and vegetation, followed by the MWA, RTE, and SCA. The SWA in the water body has the highest accuracy, and the MWA has the lowest accuracy. On the artificial surfaces, the MWA has the highest accuracy, followed by the SWA, while the SCA’s accuracy was the lowest.

Therefore, this study can provide reasonable suggestions to select LST retrieval algorithms for different land cover types in different seasons in Shanghai. MWA is more suitable for the area of cultivated land, vegetation, and artificial surfaces in winter. Simultaneously, SWA should be selected in the wetland areas, while RTE and SCA are more suitable for the water body regions. In summer, SWA is more suitable for LST retrieval in cultivated, vegetated, and the water body areas; SWA or MWA should be chosen for the wetland, while SWA is more suitable for artificial surfaces.

### 4.4. Sensitivity Analysis of the Four Algorithms

Atmospheric water vapor content, the effective mean atmospheric temperature, atmospheric transmittance, and LSE are essential parameters for LST retrieval. Since it is difficult to acquire each algorithm’s parameters, errors will inevitably occur, leading to errors in the retrieval results and impacting the algorithm’s accuracy. Thus, it is necessary to determine whether an algorithm is suitable for the study area and evaluate the influence of parameter errors on the results. Hence, the sensitivity of the different input parameters of each algorithm needs to be considered [35]. By keeping the other parameters constant when performing sensitivity analysis for a parameter, it is possible to assume no error values for the other parameters, which would allow a more realistic estimate of the land surface temperature errors. The sensitivity analysis of the parameters can be calculated by the following Formula (8):

(8) $$\Delta T_{s}=\left|T_{S}\right.\left(x+\Delta x\right)-T_{s}\left.\left(x\right)\right|$$

where ∆*x* is the possible error of parameter *x*; ∆*T_s_* is LST estimation error due to the error of parameter *x*; *T_s_(x)* and *T_s_ (x +* ∆*x)* represent the retrieved LST when the parameters are *x* and *x +* ∆*x*, respectively.

This paper analyzed four LST retrieval algorithms’ sensitivity based on Landsat-8 data from Shanghai on 21 January 2020 and 16 August 2020. The atmospheric water vapor content was 2.78 g/cm^2^ at the moment of satellite transit on 16 August 2020, and 0.64 g/cm^2^ on 21 January 2020.

#### 4.4.1. Sensitivity Analysis to Water Vapor Content

**Figure 9:**
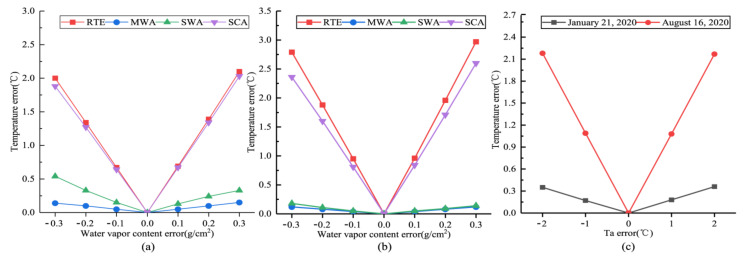
The errors in LST retrieval results due to changes in different parameters. (**a**,**b**) are the LST estimation errors resulting from the estimation errors in atmospheric water vapor content on 21 January 2020 and 16 August 2020, respectively. (**c**) is the LST estimation error of MWA due to the estimation error in effective atmosphere temperature.

Atmospheric transmittance has an essential effect on land surface thermal radiation in the atmosphere and is necessary for LST retrieval. Accurate acquisition of atmospheric transmittance improves LST retrieval accuracy. Many factors affect atmospheric transmittance, including air pressure, air temperature, aerosol content, atmospheric water vapor content, etc. The variation of atmospheric water vapor can cause apparent changes in atmospheric transmittance. Therefore, a sensitivity analysis of atmospheric water vapor content is required for each algorithm. Some scholars have indicated that air humidity error is usually smaller than ±5% [36], and the water vapor content error can be expected to be within the range of ±0.3 g/cm^2^. Therefore, the atmospheric water vapor content at the moment of satellite transit was used as the base for calculating the average LST error for each algorithm in the error range of ±0.3 g/cm^2^. The results are shown in Figure 9a,b. It can be seen from the figures that the RTE has the highest sensitivity to atmospheric water vapor content. For every 0.1 g/cm^2^ increase in water vapor content error, the LST retrieval error increases by 0.65 °C and 0.95 °C in winter and summer, respectively. The SCA’s sensitivity to atmospheric water vapor content was second only to RTE. For each 0.1 g/cm^2^ change in water vapor content error in SWA, LST retrieval error reached 0.14 °C in winter and 0.05 °C in summer. Among the four retrieval algorithms, MWA is the least sensitive to atmospheric water vapor content parameters.

#### 4.4.2. Sensitivity Analysis to Effective Atmosphere Temperature

MWA was the only one among the four algorithms to use the effective atmosphere temperature. Since the error between the temperature data recorded at the meteorological station and the effective atmospheric temperature is within 2 °C, the retrieval error of LST is calculated by increasing or decreasing by 1.0 °C or 2.0 °C, respectively, based on the moment of satellite transit. As seen in Figure 9c, on 16 August 2020, the summer has a high sensitivity to effective atmosphere temperature. When the effective atmosphere temperature error increased by 1.0 °C, the LST retrieval error reached 1.09 °C. When the maximum error reached 2 °C, the results’ error reached 2.18 °C at the maximum. Simultaneously, the sensitivity of 21 January 2020 was lower, and the retrieved temperature value error reached 0.18 °C for every 1.0 °C change in temperature error. When the maximum error of the effective atmospheric temperature was 2 °C, the maximum error value of the retrieved result was 0.36 °C.

#### 4.4.3. Analysis of Sensitivity to LSE

**Figure 10:**
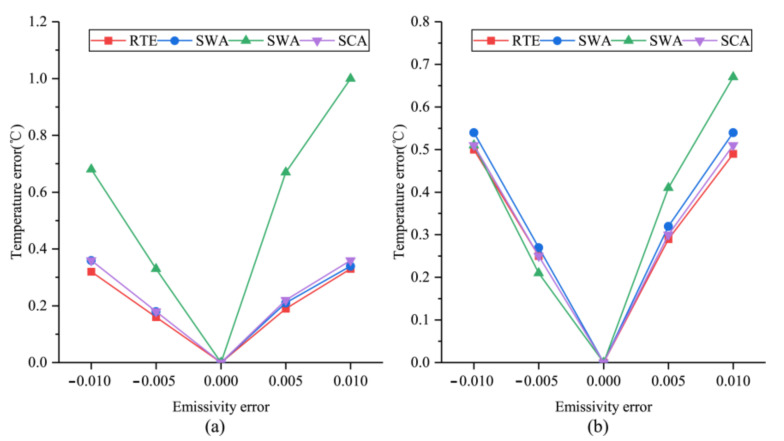
The errors in LST retrieval results due to changes in different parameters. (**a**,**b**) are the LST estimation errors of MWA due to the estimation error in effective atmosphere temperature.

LSE is another critical parameter of LST retrieval. Previous studies have shown that the estimation error of LSE is usually less than or equal to 0.006 [37], and the LSE error can be expected to be within the range of ±0.01. Therefore, this study used the experimentally calculated LSE as the base for estimating the LST error for each algorithm. The results are shown in Figure 10, and it can be seen that the SWA’s sensitivity to the LSE was the highest among the four algorithms. When the LSE error increased by 0.005, the LST retrieval error reached 0.41 °C and 0.67 °C in winter and summer, respectively. The other three algorithms’ sensitivity to LSE was approximately the same, and for every 0.005 increase in LSE error, the retrieval error was about 0.30 °C in winter and 0.20 °C in summer. 

The sensitivity analysis of the four LST retrieval algorithms leads to the following conclusions: The RTE and SCA are more sensitive to atmospheric water vapor content than the other two algorithms, and this effect will increase as the water vapor content increases. In addition, among the four LST retrieval algorithms, SWA and MWA have relatively low sensitivity to water vapor content, while the SWA has the highest sensitivity to LSE. The ability to accurately acquire the effective atmosphere temperature has a critical impact on the retrieval accuracy of the SWA.

## 5. Discussions

In this study, based on the remote sensing data of Shanghai in winter and summer provided by the Landsat-8 satellite, the four algorithms of Radiative Transfer Equation (RTE), Mono-Window Algorithm (MWA), Split-Window Algorithm (SWA), and Single-Channel Algorithm (SCA) were used in Land Surface Temperature (LST) retrieval. The results showed that the four algorithms’ spatial distribution of the urban thermal environment was basically the same. The surface temperature of artificial surfaces was significantly higher than that of the areas with high vegetation cover and near the water body. Combined with the LST profiles, the SWA could better reflect the spatial distribution pattern of Shanghai’s heat island effect than any of the other three algorithms.

The comparison of the maximum, minimum, and average values of the four algorithms’ retrieval results showed that the overall trend of the four algorithms’ results in different periods was relatively similar, but there were also differences. The differences were demonstrated explicitly when RTE and SCA used the same simultaneous atmospheric parameters, thus leading to the smallest difference in the retrieval results. However, the MWA introduced the average atmospheric temperature to approximate the upwelling and downwelling radiance in the atmosphere, which led to a larger difference between its retrieval results and those of the other three algorithms.

To further verify the LST accuracy of the four algorithms, the methods of T-based and cross-validation were employed. The measured data from meteorological stations were used in the T-based method, and the MODIS temperature products were used in the cross-validation. The results showed that all these four algorithms could be used to retrieve LST in winter with low water vapor content, and that the MWA has greater precision for LST retrieval in the area of cultivated land, vegetation, and artificial surfaces; the SWA has higher accuracy in the wetland; RTE and SCA have higher accuracy in the water body. With high water vapor content in summer, MWA and SWA are more suitable for LST retrieval in summer. SWA has higher retrieval accuracy in cultivated, vegetated, wetland, and water body areas. MWA has higher accuracy in artificial surfaces.

Comparing the four algorithms’ sensitivity analysis, it was found that RTE and SCA were more sensitive to errors in the atmospheric water vapor content, especially in hot and humid conditions. Simultaneously, the SWA increased with the water vapor content, and the water vapor content error’s effect on the results became smaller. SWA was more sensitive to the LSE than any of the other three algorithms. The MWA was sensitive to the parameter of error in effective mean atmospheric temperature, and its accurate acquisition had a critical impact on the accuracy of the retrieval.

Landsat-8 images can be used efficiently to retrieve LST due to their two thermal infrared bands. It is crucial to choose a suitable algorithm according to the different conditions in different study areas. Some reasonable recommendations can be given for combining the retrieval accuracy with the sensitivity analysis of the four algorithms. The SWA is more suitable for hot and humid summers, since RTE and SCA are more sensitive to atmospheric water vapor content errors. Meanwhile, the MWA is more sensitive to effective mean atmospheric temperature errors, making it more difficult to obtain accurate data. In low temperatures and dry winters, all four algorithms can be used to retrieve LST. However, when considering the influence of the effective mean atmospheric temperature parameter, the RTE, SWA, and SCA are recommended.

## Figures and Tables

**Table 1:** The differences (in absolute values) in the average LST values between the four algorithms for any two algorithms.

Date	RTE-MWA	RTE-SWA	RTE-SCA	MWA-SWA	MWA-SCA	SWA-SCA
2019.12.04	0.52	0.08	0.02	0.60	0.54	0.06
2020.01.21	0.92	0.95	0.05	0.03	0.97	1.00
2020.02.22	0.18	0.12	0.02	0.30	0.20	0.10
Average	0.54	0.38	0.03	0.31	0.57	0.39
2020.05.12	0.82	1.53	0.04	2.35	0.86	1.49
2019.07.29	12.58	3.72	0.87	8.86	13.45	4.59
2020.08.16	9.36	3.16	0.71	6.20	10.07	3.87
Average	7.59	2.80	0.54	5.80	8.13	3.32

RTE-MWA: the difference between RTE and MWA average surface temperature values (absolute values); The other abbreviations represent the same meaning and difference between the average surface temperature (absolute value) between the two algorithms.

## Data Availability

Data is available on request to the corresponding author.
